# Skeletal muscle phenotypic switching in heart failure with preserved ejection fraction

**DOI:** 10.3389/fcvm.2022.1016452

**Published:** 2022-12-01

**Authors:** Eng Leng Saw, Louis Dominic Werner, Payman Zamani, Julio A. Chirinos, María Valero-Muñoz, Flora Sam

**Affiliations:** ^1^Whitaker Cardiovascular Institute, Boston University School of Medicine, Boston, MA, United States; ^2^Division of Cardiovascular Medicine, Hospital of the University of Pennsylvania, Philadelphia, PA, United States; ^3^Eli Lilly and Co, Indianapolis, IND, United States

**Keywords:** heart failure with preserved ejection fraction, exercise intolerance, skeletal muscle, oxidative metabolism fibers, atrophy

## Abstract

**Background:**

Skeletal muscle (SkM) phenotypic switching is associated with exercise intolerance in heart failure with preserved ejection fraction (HFpEF). Patients with HFpEF have decreased type-1 oxidative fibers and mitochondrial dysfunction, indicative of impaired oxidative capacity. The *SAUNA* (SAlty drinking water/Unilateral Nephrectomy/Aldosterone) mice are commonly used in HFpEF pre-clinical studies and demonstrate cardiac, lung, kidney, and white adipose tissue impairments. However, the SkM (specifically the oxidative-predominant, *soleus* muscle) has not been described in this preclinical HFpEF model. We sought to characterize the *soleus* skeletal muscle in the HFpEF *SAUNA* mice and investigate its translational potential.

**Methods:**

HFpEF was induced in mice by uninephrectomy, *d*-aldosterone or saline (Sham) infusion by osmotic pump implantation, and 1% NaCl drinking water was given for 4 weeks. Mice were euthanized, and the oxidative-predominant *soleus* muscle was collected. We examined fiber composition, fiber cross-sectional area, capillary density, and fibrosis. Molecular analyses were also performed. To investigate the clinical relevance of this model, the oxidative-predominant, *vastus lateralis* muscle from patients with HFpEF was biopsied and examined for molecular changes in mitochondrial oxidative phosphorylation, vasculature, fibrosis, and inflammation.

**Results:**

Histological analyses demonstrated a reduction in the abundance of oxidative fibers, type-2A fiber atrophy, decreased capillary density, and increased fibrotic area in the *soleus* muscle of HFpEF mice compared to Sham. Expression of targets of interest such as a reduction in mitochondrial oxidative-phosphorylation genes, increased VEGF-α and an elevated inflammatory response was also seen. The histological and molecular changes in HFpEF mice are consistent and comparable with changes seen in the oxidative-predominant SkM of patients with HFpEF.

**Conclusion:**

The HFpEF *SAUNA* model recapitulates the SkM phenotypic switching seen in HFpEF patients. This model is suitable and relevant to study SkM phenotypic switching in HFpEF.

## Introduction

Heart failure with preserved ejection fraction (HFpEF) is characterized by signs and symptoms of heart failure (HF), in the presence of a normal left ventricular (LV) ejection fraction (EF) (1). HFpEF accounts for up to 50% of all clinical HF presentations and is the most common form of HF in older patients (2, 3). Evidence-based therapies for HFpEF are limited, likely due to inadequate understanding of this heterogeneous syndrome (3–6). It is also evident that, in addition to cardiac abnormalities, HFpEF involves other organ abnormalities, which likely contribute to its pathophysiology (7). As such, there is a critical need to: *(a)* understand pathophysiological changes in all organs in HFpEF and not just the heart, *(b)* utilize suitable preclinical models of HFpEF, *(c)* translate the relevance of preclinical findings to human HFpEF, and *(d)* accelerate research discoveries to facilitate the development of novel HFpEF therapies (7).

Exercise intolerance and limitations in aerobic capacity are central features of HFpEF (8, 9). Prior studies suggest that, in addition to cardiac dysfunction, non-cardiac peripheral factors including skeletal muscle (SkM) phenotypic switching, contribute to the reduced physical activity present in HFpEF patients (10–16). Similarly, SkM myopathy and dysfunction are also observed in comorbidities that are commonly seen in HFpEF patients such as hypertension (17), chronic obstructive pulmonary disease (18, 19), obesity (20, 21), chronic kidney disease (22), diabetes mellitus (23, 24), etc. As such, these extracardiac comorbidities may also contribute to the SkM phenotype seen in these patients and to the overall poor outcome (25). SkM phenotypic switching includes, among others, muscle atrophy (26, 27), fiber composition shift (16, 28), decreased capillary density (28) and altered secretion of SkM-derived factors (29–31). While this suggests that a SkM-directed approach might be beneficial, the development of targeted therapies is hindered by the lack of preclinical models that recapitulate the SkM phenotypic switching seen in HFpEF patients (32).

The skeletal muscle is a heterogenous bundle of muscle fiber types. Type-1 (“slow-twitch”) oxidative fibers express myosin heavy chain (*MYH*) 7 and are enriched with mitochondria primarily utilizing oxidative metabolism to generate ATP (33, 34). Conversely, the “fast-twitch” fibers, type-2X (*MYH1*) and type-2B glycolytic fibers (*MYH4*), have less mitochondrial content and predominantly use glycolysis for metabolism. Finally, the type-2A oxidative fibers are also considered “fast twitch” (*MYH2*), but they utilize oxidative metabolism and have intermediate contractile properties between type-1 and type-2X/type-2B. Human SkM has three major fiber types (i.e., type-1, type-2A and type-2X), while in mouse SkM, there are four major types (i.e., type-1, type-2A, 2X and 2B fibers) (33, 34). Both *vastus lateralis* and *soleus* muscle contain high numbers of type-1 and type-2A oxidative fibers and are thus predominantly oxidative (33–36) (Supplementary Figure 1).

We, and others, previously demonstrated that the HFpEF *SAUNA* (SAlty drinking water/Unilateral Nephrectomy/Aldosterone) mouse model is characterized by exercise intolerance and pulmonary congestion in addition to cardiac (i.e., LV hypertrophy, diastolic dysfunction with a preserved LVEF) (37–40) and extra-cardiac abnormalities (i.e., renal and white adipose tissue dysfunction) (37, 38, 41–44). However, the oxidative-predominant SkM (the *soleus* muscle) of this model has not been characterized. Therefore, we sought to investigate the *soleus* muscle phenotype in HFpEF mice and establish the clinical relevance of these findings in human HFpEF by utilizing SkM biopsies from the oxidative-predominant *vastus lateralis* muscle and biomarkers obtained from patients with HFpEF.

## Materials and methods

All data that support the findings of this study, including methods and study materials, are available from the corresponding authors upon reasonable request.

*An expanded Materials and Methods section is available in the Supplementary material.*

### Ethical approval

The Institutional Animal Care and Use Committee at Boston University School of Medicine approved all procedures related to the handling and surgery of the mice. The University of Pennsylvania and Boston University Medical Campus Institutional Review Boards approved the human studies, including human *vastus lateralis* muscle biopsies and human serum samples, respectively. All subjects provided written informed consent prior to entry into the study.

### Human *vastus lateralis* muscle biopsy from UPenn HFpEF cohort

Using the suction-modified Bergstrom technique (45), percutaneous biopsy of the *vastus lateralis* muscle was performed in HFpEF patients and control subjects without a history of HFpEF. The HFpEF patients had New York Heart Association (NYHA) class II/III symptoms, an LVEF ≥ 50%, and were on stable medical therapy for at least one month. See inclusion and exclusion criteria for the human HFpEF patients in the Supplementary material.

### HFpEF *SAUNA* murine model

Eight-week-old C57BL/6J mice (Jackson Laboratories) underwent HFpEF induction using the *SAUNA* model. Mice were anesthetized with 80 mg/kg ketamine and 8 mg/kg xylazine intraperitoneally. All mice underwent uninephrectomy and then received either a continuous infusion of 0.9% normal saline (Sham) or *d*-aldosterone (0.30 μg/h; Sigma-Aldrich, A9447; HFpEF) for 4-weeks via osmotic minipumps (Alzet, 1004). All mice were also given 1.0% sodium chloride drinking water. At 4-weeks, mice were sacrificed and the *soleus* muscle [i.e., oxidative-type fibers predominant (35)] was collected for further analyses.

### Hemodynamic measurements

Heart rate (HR), systolic and diastolic blood pressures (BP) of HFpEF mice and Sham were measured at the end of the 4-weeks using a non-invasive tail-cuff BP-2000 blood pressure analyzer (Visitech Systems).

### Immunofluorescent analysis

To examine the fiber composition, immunofluorescence analysis of myosin heavy chain (MHC) isoforms was performed by probing the mice muscle sections with primary antibodies against MHCI (i.e., type-1 slow twitch/oxidative fiber), MHCIIa (i.e., type-2A fast twitch/oxidative fiber), MHCIIx (type-2X fast twitch/glycolytic fiber) and dystrophin (SkM-specific marker). Unstained fibers were identified as type-2B fast twitch/glycolytic fiber and fibers co-expressing MHCI and MHCIIa were identified as type-1/2A hybrid fiber. (See list of antibodies in Supplementary material). The number of each specific fiber type was expressed as the mean number of fibers per total number of fibers as a percentage. The cross-sectional area of each specific fiber type was expressed as the mean area in μm^2^.

To examine the density of capillaries, sections were probed with Isolectin GS-IB_4_, to detect endothelial cells, and primary antibody against the skeletal muscle specific marker, dystrophin. The capillary density was expressed as the mean number of Isolectin^+^ cells per total number of fibers. (See expanded method in Supplementary material).

### Picrosirius red analysis

To examine fibrosis, mice muscle sections were first stained with 0.04% Fast Green (Sigma-Aldrich, F7258) in 1.3% saturated picric acid (Sigma-Aldrich, 197378), and followed by 0.1% Fast Green/0.04% Sirius red (Sigma-Aldrich, 365548) in 1.3% saturated picric acid (46). The fibrotic area was normalized to total tissue area and expressed as the fold change as compared to Sham mice.

### Gene expression analysis

Total RNA was extracted from mice *soleus* muscle and human *vastus lateralis* muscle according to manufacturer’s instructions. After reverse transcription, quantitative polymerase chain reaction (qPCR) was performed using a ViiA7 PCR system (Applied Biosystem). Details of mouse-specific and human-specific primers are provided in the Supplementary material (Supplementary Tables 1, 2).

### Western blot analysis

Proteins were extracted from *soleus* muscle in mice and human *vastus lateralis* muscle using ice-cold RIPA buffer, subjected to sodium dodecyl sulfate (SDS)-polyacrylamide gel electrophoresis (PAGE), and transferred onto a polyvinylidene difluoride (PVDF) membrane prior incubation with specific antibodies. Details of primary and secondary antibodies used are provided in the Supplementary material (Supplementary Table 3). The band intensity of protein of interest was normalized to the band intensity of α-/β-tubulin and expressed as the fold change compared to Sham mice or the human control group. Uncropped blots showed in main figures are included in the Supplementary Figure 4.

### Serum VEGF-α analysis

Circulating levels of total vascular endothelial growth factor-alpha (VEGF-α) were measured from serum samples from a second cohort of chronic ambulatory patients from the Boston Medical Center (BMC) HFpEF cohort and control subjects. See inclusion and exclusion criteria in the Supplementary material. Enzyme-linked immunoassay was performed according to manufacturer’s instructions (R&D systems). The protein concentration was extrapolated using a standard curve generated with recombinant standards provided by the manufacturer and expressed as pg/mL.

### Statistical analysis

All statistical analyses were performed using GraphPad Prism software. Normal distribution of data was verified by D’Agostino & Pearson omnibus normality test. Differences between the two groups were tested by unpaired *t* test or the Mann-Whitney *U* test, as dictated by the distribution. Data was expressed as mean ± standard error of the mean (SEM) for mice, mean ± standard deviation (SD) or median (interquartile range) for humans. A *P*-value < 0.05 was considered as statistically significant. For details of the power calculations for mice and human HFpEF cohorts, see the Supplementary material section for extended methods.

## Results

### HFpEF mice

Similar to prior studies (37, 38, 41–44), HFpEF *SAUNA* mice demonstrated moderate hypertension with an elevated systolic BP (135.6 ± 2.9 *vs.* 114.4 ± 3.0 mm Hg; *P* < 0.001) and increased diastolic BP (110.4 ± 2.8 *vs.* 88.0 ± 2.7 mm Hg; *P* < 0.0001) *vs.* Sham. HFpEF mice had increased lung congestion as quantified by an increased wet-to-dry lung ratio (4.5 ± 0.0 *vs.* 4.3 ± 0.1; *P* < 0.01). Cardiac hypertrophy, measured as an increased heart weight-to-tibia length ratio, was seen in HFpEF mice *vs.* Sham (77.7 ± 3.3 *vs.* 67.2 ± 2.6 mg/cm; *P* < 0.05; Table 1).

**TABLE 1:** HFpEF animal characteristics (after 4 weeks of *SAUNA* HFpEF induction).

	HFpEF (*n* = 11)	Sham (*n* = 7)	*P*-value
Systolic blood pressure (mm Hg)	135.6 ± 2.9	114.4 ± 3.0	< 0.001
Diastolic blood pressure (mm Hg)	110.4 ± 2.8	88.0 ± 2.7	< 0.0001
Heart rate (beats/min)	513.8 ± 21.2	619.4 ± 23.4	< 0.01
Tibia length (cm)	2.1 ± 0.0	2.1 ± 0.0	N.S.
Wet-to-Dry lung weight ratio	4.5 ± 0.0	4.3 ± 0.1	< 0.01
Heart weight-to-Tibia length ratio	77.7 ± 3.3	67.2 ± 2.6	< 0.05
Gastrocnemius muscle weight/Tibia length (mg/cm)	51.3 ± 1.9	64.1 ± 2.3	< 0.001
Soleus muscle weight/Tibia length (mg/cm)	4.3 ± 0.1	5.4 ± 0.1	< 0.0001

All data are presented as mean ± SEM. Statistical analysis by Student *t* test for normally distributed data or Mann-Whitney *U* test for non-distributed data.

Notably, HFpEF mice showed calf muscle atrophy characterized by a significant reduction in the weight of both *soleus* muscle relative to tibia length (4.3 ± 0.1 *vs.* 5.4 ± 0.1 mg/cm; *P* < 0.0001) and *gastrocnemius* muscle (51.3 ± 1.9 *vs.* 64.1 ± 2.3 mg/cm; *P* < 0.001) *vs.* Sham; Table 1.

### Skeletal muscle fiber composition and cross-sectional area in HFpEF mice

**FIGURE 1:**
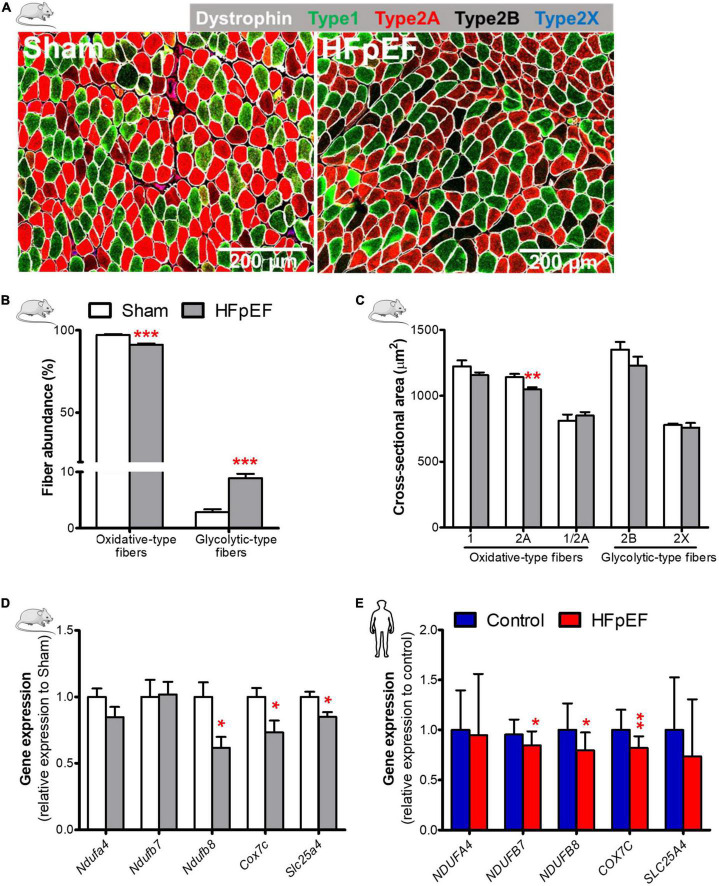
Decreased oxidative fiber, fiber atrophy, and downregulation of mitochondrial oxidative genes in HFpEF. **(A)** In mice, representative confocal images showing staining of dystrophin (white) and type-1 (green), type-2A (red), type-2X (blue) fibers in *soleus* muscle. **(B)** Quantitative analysis showing the abundance of oxidative-type and glycolytic-type fibers, and **(C)** the cross-sectional area size of each individual fiber type in Sham and HFpEF mice (*n* = 7-11/group). **(D)** Quantitative analysis of the relative expression of mitochondrial oxidative genes – *Ndufa4*, *Ndufb7*, *Ndufb8*, *Cox7c* and *Slc25a4* in Sham (*n* = 5-7) and HFpEF mice (*n* = 6-15). **(E)** In humans, quantitative analysis of relative gene expression of *NDUFA4*, *NDUFB7*, *NDUFB8*, *COX7C* and *SLC25A4* in *vastus lateralis* muscle of control (*n* = 12-13) and HFpEF patients (*n* = 10). Mice and human data are presented as mean ± SEM and mean ± SD, respectively. Statistical analysis by Student *t* test for normally distributed data or Mann-Whitney *U* test for non-distributed data. *P < 0.05, **P < 0.01, ***P < 0.001 *vs.* Sham mice or control subjects. NADH: ubiquinone oxidoreductase (NDU) subunit A4, Ndufa4; NDU subunit B7, Ndufb7; NDU subunit B8, Ndufb8; Cytochrome C oxidase subunit 7C, Cox7c; Adenine nucleotide translocator 1, Slc25a4.

Immunofluorescence analysis of the predominantly oxidative *soleus* muscle in HFpEF mice showed a significant reduction in the abundance of the oxidative-type fibers [combined type-1, type-2A and type-1/2A hybrid fibers (34)] 91.2 ± 0.8 *vs.* 97.2 ± 0.5%; *P* < 0.0001; and a significant increase in the glycolytic-type fibers [combined type-2B and type-2X (34)] 8.9 ± 0.8 *vs.* 2.8 ± 0.5%; *P* < 0.0001 (Figures 1A,B and Supplementary Figure 2) as compared to Sham mice.

The cross-sectional area of type-2A, the most abundant fiber type in *soleus* muscle in mice (∼61%, Supplementary Figure 2), was significantly reduced in HFpEF mice (1,050.0 ± 14.8 *vs.* 1,141.0 ± 24.5 μm^2^; *P* < 0.01) as compared to Sham, but there were no significant changes in type-1, type-2B, type-2X or type-1/2A hybrid fibers (Figure 1C) between HFpEF mice *vs.* Sham. Thus, the major contributor to *soleus* muscle atrophy is a reduction in the size of the more abundant type-2A fibers in HFpEF mice (Table 1).

### Oxidative phosphorylation gene expression in HFpEF mice and the UPenn HFpEF patients

Accompanying the reduction in the oxidative-type fibers of the *soleus* muscle in HFpEF mice was a significant downregulation of specific genes involved in the electron transport system where NADH:ubiquinone oxidoreductase (NDU) subunit B8 (*Ndufb8*) was reduced by 39%, cytochrome C oxidase subunit 7C (*Cox7c*), was reduced by 27%, and adenine nucleotide translocator 1 (*Slc25a4*) was reduced by 15% (P < 0.05 for all) respectively, in HFpEF mice *vs.* Sham (Figure 1D).

Similarly, we measured the expression of mitochondrial oxidative genes in the SkM biopsy samples obtained from the UPenn HFpEF patients. See Table 2 for detailed patient characteristics.

**TABLE 2:** Characteristics of the UPenn cohort of chronic stable HFpEF patients from whom skeletal muscle biopsy was obtained.

	Chronic HFpEF (*n* = 12)	Control (*n* = 13)	*P*-values	Median (IQR) for HFpEF	Median (IQR) for control
**Clinical characteristics**					
Age, years	64.7	54.6	<0.05	67.0 (59.3-76.5)	57.0 (48.0-67.5)
Sex: Male/Female (%)	6/6 (50/50)	9/4 (69/31)			
Race: White/Black/Other (%)	7/5/0 (58/42/0)	13/0/0 (100/0/0)			
Body mass index (BMI; kg/m^2^)	37.2 ± 8.4	26.5 ± 3.4	<0.001		
Body surface area (BSA; m^2^)^a^	2.2 ± 0.3	2.0 ± 0.2	<0.01		
**Comorbidities (%)**					
Hypertension	11 (92)	−			
Obesity (BMI > 30 kg/m^2^)	9 (75)	2 (15)			
Type 2 diabetes mellitus	8 (67)	−			
Prior history of atrial fibrillation/Flutter	4 (33)	−			
Coronary artery disease	3 (25)	−			
NYHA functional class II/III (%)	10/2 (83/17)	−			
Heart rate, beats/min	65.0 ± 9.0	62.7 ± 10.0	N.S.		
**Echocardiographic parameters**					
LVEF,%	61.4 ± 5.9	60.8 ± 7.0	N.S.		
IVS, cm	1.0 ± 0.2	0.9 ± 0.2	N.S.		
PW, cm	1.0 ± 0.2	0.8 ± 0.1	<0.05		
Calculated LV mass^b^, g	171.4 ± 44.0	132.0 ± 37.7	<0.05		
Calculated LV mass/BSA, g/m^2^	77.3 ± 16.4	67.3 ± 16.4	N.S.		
LVEDD, cm	4.7 ± 0.6	4.6 ± 0.3	N.S.		
LVESD, cm	3.0 ± 0.5	3.1 ± 0.4	N.S.		
Relative wall thickness	0.5 ± 0.1	0.4 ± 0.07	N.S.		
Mitral E velocity, cm/s	84.9 ± 16.6	68.1 ± 14.8	<0.05		
Mitral A velocity. cm/s	76.6	51.0	<0.01	79.3 (65.9-83.9)	50.2 (42.4-59.4)
Mitral E/A ratio	1.3	1.4	N.S.	1.2 (0.8-1.4)	1.4 (1.1-1.5)
Mitral E/septal e’ ratio	11.6	7.0	<0.01	12.5 (8.6-13.6)	6.5 (5.7-8.0)
**Biomarkers**					
NT-proBNP, pg/ml	137.5 ± 111.8	55.4 ± 33.3	<0.05		
Creatinine, mg/dl	1.0 ± 0.3	0.9 ± 0.2	N.S.		
CKD-EPI eGFR, mL/min	73.8	85.3	N.S.	73.9 (61.9-91.5)	88.8 (81.0-93.2)
Sodium, mmol/L	138.2 ± 3.3	138.5	N.S.	138.0 (137.0-140.0)	138.0 (137.0-140.5)
Glucose, mg/dl	121.7 ± 31.9	89.7 ± 6.4	<0.01		
Potassium, mmol/L	4.1	4.3	N.S.	4.2 (3.7-4.4)	4.2 (4.0-4.4)
**Medications (%)**					
Diuretics (Loop/Thiazide)	7 (58)	0 (0)			
Mineralocorticoid receptor antagonist	3 (25)	0 (0)			
ACE inhibitor/ARB	9 (75)	0 (0)			
β-blockers	9 (75)	0 (0)			
Calcium channel blockers	6 (50)	0 (0)			
Anticoagulant	3 (25)	0 (0)			
Statin	7 (58)	3 (23)			
Aspirin/Antiplatelet	9 (69)	5 (38)			
Insulin	3 (25)	0 (0)			

All data are expressed as mean ± SD for continuous variables or numbers or percent (%) for categorical variables, or median and interquartile range (for non-normally distributed data). Statistical analysis by Student *t* test for normally distributed data or Mann-Whitney *U* test for non-distributed data. LV, left ventricular; LVEF, left ventricular ejection fraction; IVS, Intraventricular septal thickness; LVEDD, LV end diastolic diameter; LVESD, LV end systolic diameter; PW, posterior wall thickness; NYHA, New York Heart Association; IQR, interquartile range; CKD-EPI eGFR, estimated glomerular filtration rate by Chronic Kidney Disease Epidemiology Collaboration creatinine equation; NT-proBNP, N-terminal pro-brain natriuretic peptide; ACE, angiotensin-converting enzyme; ARB, angiotensin II receptor blocker. ^a^Body surface area was calculated based on the Gehan and George formula “Weight (kg)^0.51456^ x Height (cm)^0.4.2246^ x 0.0235) m^2^” (81). ^b^LV mass was calculated based on formula “0.8 × 1.04 x ([LVEDD + PW + IVS]^3^ – [LVEDD]^3^) + 0.6 g” per guideline recommendations by the American Society of Echocardiography (82).

Briefly, *HFpEF patients* from the UPenn cohort were ambulatory, *chronic ambulatory stable* and predominantly NYHA Functional class II and III. Comorbidities included hypertension (92%), obesity (75%), type 2 diabetes mellitus (T2DM; 67%), atrial fibrillation/flutter (33%) and coronary artery disease (25%). Echocardiography demonstrated preserved LVEF (61.4 ± 5.9%) with evidence of adverse cardiac remodeling including increased mean posterior wall (PW) thickness (1.0 ± 0.2 cm), calculated LV mass (171.4 ± 44.0 g) and relative wall thickness (RWT; 0.47 ± 0.11). There was evidence of diastolic dysfunction, as indicated by the increased E/A ratio (1.3 ± 0.7) and mitral E/septal e’ ratio (11.6 ± 3.2).

*Controls subjects* were healthy, with a mean age of 55 ± 17 years and 15% were obese with a body mass index (BMI) > 30 kg/m^2^. No control subjects had hypertension and 23% and 38% were on a statin and aspirin, respectively. Echocardiography was normal in the control subjects with no evidence of adverse cardiac remodeling nor diastolic dysfunction.

Gene analysis of the SkM biopsies obtained from these HFpEF patients showed that *NDUFB7*, *NDUFB8* and *COX7C* expression were decreased in HFpEF patients by 11% (*P* < 0.05), 21% (*P* < 0.05) and 18% (*P* < 0.01), respectively *vs.* control subjects (Figure 1E).

### Skeletal muscle capillary density in HFpEF mice and the UPenn and BMC HFpEF patient cohorts

**FIGURE 2:**
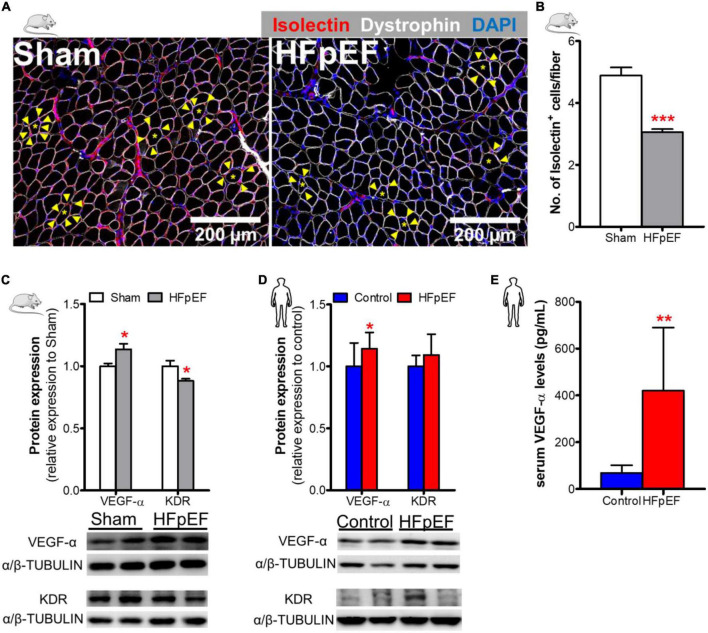
Decreased SkM capillary density in HFpEF. **(A)** In mice, representative confocal images showing staining of dystrophin (white), isolectin-stained endothelial cells (red; indicated by yellow arrows) and nuclei (blue) in *soleus* muscle. **(B)** Quantitative analysis showing the capillary density in Sham and HFpEF mice (*n* = 7-11/group). **(C)** Representative blots and quantitative analysis of relative protein expression of VEGF-α and KDR in Sham and HFpEF mice (*n* = 7-11/group). **(D)** In humans, representative blots, and quantitative analysis of relative protein expression of VEGF-α and KDR in the *vastus lateralis* muscle of control (*n* = 12-13) and HFpEF patients (*n* = 11-12). **(E)** Quantitative analysis of circulating VEGF-α levels in control (*n* = 4) and HFpEF patients (*n* = 20). Statistical analysis by Student *t* test. Mouse and human data are presented as mean ± SEM and mean ± SD, respectively. *P < 0.05, **P < 0.01, ***P < 0.001 *vs.* Sham mice or control subjects. VEGF-α, Vascular endothelial growth factor-α; KDR, Kinase insert domain receptor.

To measure the capillary density of SkM of HFpEF mice, immunofluorescence analysis was performed which showed that the number of isolectin-stained endothelial cells per fiber was reduced in the *soleus* muscle of HFpEF mice as compared to Sham (3.1 ± 0.1 *vs.* 4.9 ± 0.3; *P* < 0.0001; Figures 2A,B). Additional studies showed that the pro-angiogenic VEGF-α protein expression was increased by 14% and kinase insert domain receptor (KDR, type-2 VEGF receptor) protein expression was decreased by 12% in the *soleus* muscle of HFpEF mice compared to Sham (*P* < 0.05 for both; Figure 2C).

These findings were then compared to VEGF-α and KDR protein expression in the human SkM biopsies from HFpEF patients. VEGF-α expression was similarly increased by 14% in HFpEF patients (*P* < 0.05 *vs.* control; Figure 2D). However, KDR protein expression was unchanged between HFpEF patients and controls.

Similar, to the UPenn HFpEF patients from whom the SkM muscle biopsies were obtained, blood samples were obtained from an additional *chronic, ambulatory stable HFpEF* cohort followed at an ambulatory HF Clinic at Boston Medical Center (BMC) to measure serum VEGF-α concentrations. The mean age of these HFpEF patients was 64 ± 7 years, 60% were female and 65% black. The mean BMI was 39 ± 8 kg/m^2^. Systolic BP was 133.0 ± 25.0 mmHg and diastolic BP was 77.0 ± 9.0 mmHg. Ninety-seven percent of HFpEF patients had hypertension and 62% had T2DM. LVEF was 64.0 ± 6.0%; left atrial size was increased (4.5 ± 7.0 cm) as was the mean RWT (0.5 ± 0.2) and mean LV mass (200.2 ± 54.4 grams). Diastolic dysfunction (I-III) was evident in 72% of these patients. Control subjects were healthy individuals without cardiac disease and were not taking cardiovascular medications; mean age was 57 ± 13 years and 50% were female. Circulating serum VEGF-α concentrations were significantly increased (419.6 ± 270.3 *vs.* 68.3 ± 33.2 pg/mL; *P* < 0.01; Figure 2E) in HFpEF *vs.* control subjects (who had no history of HFpEF nor cardiac disease).

### Skeletal muscle fibrosis in HFpEF mice and UPenn HFpEF patients

**FIGURE 3:**
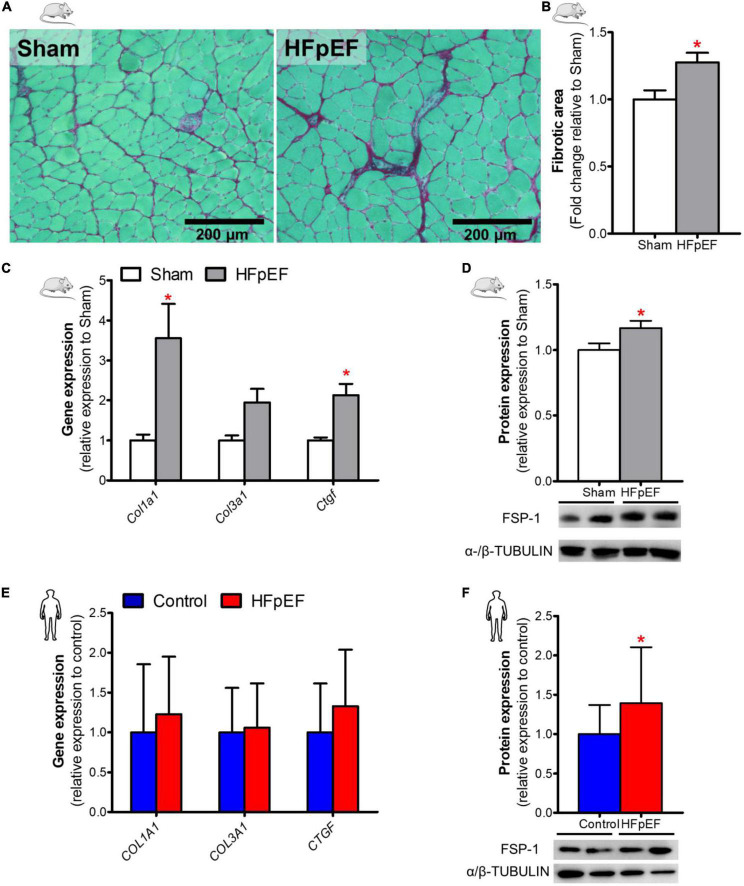
Increased SkM fibrosis in HFpEF. **(A)** In mice, representative brightfield images and **(B)** Quantitative analysis showing the fibrotic area in *soleus* muscle of Sham and HFpEF mice (*n* = 5-9/group). **(C)** Quantitative analysis of relative gene expression of *Col1a1*, *Col3a1* and *Ctgf* in Sham (*n* = 6-7) and HFpEF mice (*n* = 11-15). **(D)** Representative blots and quantitative analysis of relative protein expression of FSP-1 in Sham and HFpEF mice (*n* = 7-11/group). **(E)** In humans, quantitative analysis of relative gene expression of *COL1A1*, *COL3A1* and *CTGF* in *vastus lateralis* muscle of control (*n* = 12-13) and HFpEF patients (*n* = 10-11). **(F)** Representative blots and quantitative analysis of relative protein expression of FSP-1 in control (*n* = 13) and HFpEF patients (*n* = 12). Statistical analysis by Student *t* test for normally distributed data or Mann-Whitney *U* test for non-distributed data. Mouse and human data are presented as mean ± SEM and mean ± SD, respectively. *P < 0.05 *vs.* Sham mice or control subjects. Col1a1, Collagen type-I alpha 1 chain; Col3a1, collagen type-III alpha 1 chain; Ctgf, connective tissue growth factor; FSP-1, Fibroblast specific protein-1.

Fibrosis, as measured by picrosirius red staining, in the *soleus* muscle of HFpEF mice was significantly increased by 28% as compared to Sham (*P* < 0.05; Figures 3A,B). Similarly, collagen type-I alpha 1 chain (*Col1a1*) and the pro-fibrotic connective tissue growth factor (*Ctgf*) gene expression were both increased in HFpEF mice by 3.6- and 2.1-fold, respectively in HFpEF mice *vs.* Sham (*P* < 0.05, for both), although collagen type-III alpha 1 chain (*Col3a1*) gene expression was no different between HFpEF mice *vs.* Sham (Figure 3C). Additionally, protein expression by western blot analysis demonstrated that the myofibroblast marker fibroblast-specific protein-1 (FSP-1) (47, 48) was significantly increased by 17% (P < 0.05) in HFpEF mice *vs.* Sham (Figure 3D).

In the human SkM biopsies, gene expression of *COL1A1*, *COLA3A1* and *CTGF* were no different between HFpEF patients and control subjects (Figure 3E). However, similar to HFpEF mice, FSP-1 protein expression was significantly increased by 39% (*P* < 0.05) in HFpEF patients vs. control subjects (Figure 3F).

### Skeletal muscle inflammation in HFpEF mice and UPenn HFpEF patients

**FIGURE 4:**
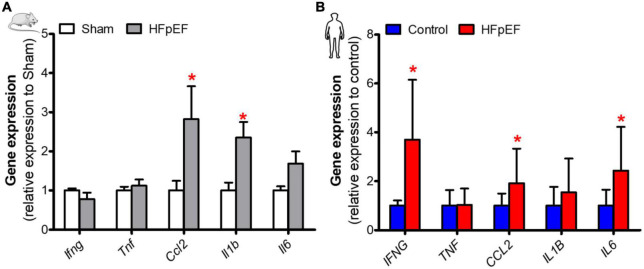
Elevated expression of inflammatory mediators in SkM in HFpEF. **(A)** In mice, quantitative analysis of relative gene expression of *Ifng*, *Tnf*, *Ccl2*, *Il1b*, *Il6* in *soleus* muscle of Sham (*n* = 6-7) and HFpEF mice (*n* = 9-15). **(B)** In humans, quantitative analysis of relative gene expression of *IFNG*, *TNF*, *CCL2*, *IL1B*, *IL6* in *vastus lateralis* muscle of control (*n* = 4-13) and HFpEF patients (*n* = 7-11). Statistical analysis by Student *t* test for normally distributed data or Mann-Whitney *U* test for non-distributed data. Mouse and human data are presented as mean ± SEM and mean ± SD, respectively. *P < 0.05 *vs.* Sham mice or control subjects. Ifng, Interferon-gamma; Tnf, Tumor necrosis factor; Ccl2, C-C motif chemokine ligand 2; Il1b, Interleukin 1-beta; Il6, Interleukin 6.

Cytokine expression was assessed to determine its association with SkM phenotypic switching in HFpEF. In the *soleus* muscle of HFpEF mice, the C-C motif chemokine ligand 2 (*Ccl2*) and interleukin 1-beta (*Il1b*) gene expression were both significantly increased by 2.8- and 2.4-fold, respectively, as compared to Sham (*P* < 0.05 for both; Figure 4A). Interferon-gamma (*Ifng*), tumor necrosis factor (*Tnf*) and interleukin 6 (*Il6*) gene expression were no different between HFpEF mice and Sham.

In the SkM biopsies from the patients with HFpEF, *IFNG*, *CCL2*, and *IL6* gene expression were significantly increased by 3.7-, 1.9- and 2.4-fold, respectively, *vs.* controls (*P* < 0.05 for all; Figure 4B). *IL1B* and *TNF* gene expression were no different between HFpEF patients and control subjects.

## Discussion

In the present study, we demonstrate phenotypic switching in the SkM of the *SAUNA* mouse model of HFpEF with a reduction in oxidative-type fibers, an increase in glycolytic-type fibers, fiber atrophy, decreased capillary density, increased fibrosis, and an elevated inflammatory response. The molecular changes in HFpEF mice are consistent with the changes seen in human SkM biopsies and serum biomarkers obtained from patients with HFpEF, demonstrating that the *SAUNA* HFpEF mouse model recapitulates the SkM phenotype seen in patients with HFpEF. Nevertheless, the human data should be interpreted cautiously as the controls and HFpEF human patients differed according to age, sex, race, and BMI: all potential cofounders which may influence the muscle fiber phenotype and activity. The limitations of obtaining muscle biopsies are previously noted, as this is an invasive procedure as well as contra-indications related to comorbidities and concurrent medications in an older population and anticipated high risk for complications (49), adding to the difficulty of adequately enrolling control subjects for an invasive procedure with no clinical justification. However, it must be noted that the primary aim of including these human samples was to correlate them with preclinical findings obtained from the *SAUNA* model aiding in the translational relevance of this HFpEF model.

The pathophysiology of HFpEF is complex as it often involves impaired function of other organs in addition to the heart (7). Although there is no perfect preclinical HFpEF model, an “ideal” pre-clinical model of HFpEF should mimic the common features of human HFpEF including the cardiac, hemodynamic, neurohormonal and peripheral abnormalities (50–53). Most recently sodium-glucose cotransporter-2 (SGLT-2) inhibitors were the *first-in-class* therapy to show a benefit in human HFpEF with improvements in HF hospitalization and cardiovascular mortality (6). SGLT-2 inhibitors alter the metabolic milieu, improve renal function, have effects on ion-channels, mediate anti-inflammatory, and anti-oxidative effects amongst others, demonstrating that benefits *outside* of the heart may indeed have beneficial effects in HFpEF (54). Thus, it is important to phenotype pre-clinical models of HFpEF to determine their relevance to the human disease, and to consider that SkM-directed approaches in selected HFpEF phenotypes. Hypertension remains *the* most common comorbidity in HFpEF patients (> 90% in HFpEF trials) and is implicated in both the pathogenesis and prognosis of HFpEF (3–6). We thus utilized the *SAUNA* model, a murine model of chronic, moderate hypertension associated HFpEF, to examine the SkM phenotype in HFpEF.

Prior studies report that abnormal oxygen extraction and utilization occurs in SkM, as evidenced by a decreased arterio-venous oxygen content difference and is a major contributor to reduced peak exercise oxygen uptake (peak VO_2_) in HFpEF patients with exercise intolerance (10–16). Furthermore, reduced SkM oxidative capacity, reduced type-1 oxidative fibers, reduced mitochondrial content and mitochondrial dysfunction all correlate with exercise intolerance in HFpEF patients (16, 28, 55). We and others previously reported that HFpEF *SAUNA* mice exhibit exercise intolerance, as shown by an inability to sustain running on a treadmill (43, 44). In the present study, oxidative-type fibers [i.e., both type-1 and type-2A fibers (34)] were reduced, accompanied by a decrease in the genes involved in mitochondrial oxidative phosphorylation in the *soleus* muscle of HFpEF mice. The downregulation of mitochondrial oxidative genes was similarly observed in human SkM biopsies obtained from patients with HFpEF, mirroring the decreased type-1 oxidative fibers seen by Zamani et al. (16) in the same cohort of patients.

In addition, the abundance of type-2B glycolytic fibers was increased 3.4-fold in the *soleus* muscle in HFpEF mice as compared to Sham (Supplementary Figure 2). Although type-2B fibers are not present in human SkM, these findings are comparable to those of human SkM biopsies from HFpEF patients reported by Zamani et al. where an increased in type-2X glycolytic fibers in the *vastus lateralis* muscle was seen (16). Type-2X are the only glycolytic-type fibers present in human SkM (34, 56). In the present study, both *soleus* and *gastrocnemius* muscle of HFpEF mice demonstrated atrophy, which is also consistent with human findings reported by Haykowsky et al. (57). In that study, HFpEF patients demonstrated atrophy with a decrease percentage of thigh SkM relative to total thigh area as measured by magnetic resonance imaging. While the SkM changes in HFpEF mice are consistent with human HFpEF, the mechanistic underpinnings of these changes are unknown.

Impaired arterial delivery of blood to exercising muscle has been demonstrated in HFpEF patients, with decreased vasodilatory reserve and reduced blood flow responses to isolated muscle during exercise (58–60). We speculate that this may activate hypoxic signaling via hypoxia-inducible factor-1 alpha (HIF-1α) in both HFpEF mice and humans with HFpEF (Supplementary Figures 5A,B). Lunde et al. (61) previously demonstrated that *in vivo* and *in vitro* overexpression of *HIF-1*α gene in electrically-stimulated SkM of rats for 14 days and in C2C12 myotubes caused MHC isoforms changes and a switch from a slow-to-fast fiber type, suggesting that HIF-1α or a lack of oxygen itself may trigger muscle changes. Moreover, HIF-1 has been shown to impair mitochondrial biogenesis and cellular respiration in other models (62). This was not investigated further as it is beyond the scope of our present study. Nevertheless, additional studies are needed to investigate the relationship between hypoxic signaling and slow-to-fast fiber type switching in HFpEF.

In addition to impaired vasodilation discussed above, a reduction in capillary density is seen with SkM phenotypic switching in HFpEF patients (28). In the present study, decreased capillary density was seen with the decreased KDR expression in the *soleus* muscle of HFpEF mice. KDR is predominantly expressed in the vascular endothelial cells and mediates signaling to promote angiogenesis (63, 64). The decreased KDR expression in HFpEF mice was accompanied by a significant increase in VEGF-α expression, which may be a compensatory response to receptor downregulation. Although SkM biopsies from HFpEF patients did not show differences in capillary-to-fiber ratio and capillary density [data published in supplemental results by Zamani et al. (16)] or KDR expression in our present study, we found VEGF-α was upregulated in *both* human SkM biopsies and serum samples from HFpEF patients. These results are consistent with HFpEF mice *and* the PROMIS-HFpEF human study where elevated circulating VEGF-α levels were seen in a large cohort of chronic HFpEF patients (31). Although VEGF-α may have angiogenic and regenerative properties in SkM (65), VEGF-α itself may increase alpha-smooth muscle actin and fibronectin expression in fibroblasts derived from the diaphragm and gastrocnemius muscle (66). Thus, chronically increased VEGF-α may underlie HFpEF pathogenesis but this proposed mechanism requires further study.

A common theme to the many comorbidities seen in HFpEF is also inflammation (38, 67, 68). Circulating inflammatory mediators such as IL-6, TNF-α, and C-reactive protein are increased and correlate to the severity of human HFpEF (29–31). In the context of SkM, prior studies showed that IL-6, IL-1β, TNF-α, IFN-γ, CCL2, IL-8 expression were increased in the *vastus lateralis* muscle from chronic, stable *HFrEF* patients (69, 70) and also in older subjects with reduced peak VO_2_ (71). The latter suggests that increased inflammation in SkM may also play a role in exercise intolerance in HFpEF. In our study, inflammatory mediators were increased in the SkM of HFpEF mice and in patients with HFpEF. While the mechanistic role of these mediators in SkM in HFpEF is unknown, chronic exposure to IL-6 has been shown to induce SkM atrophy (72, 73).

SkM fibrosis is observed in aging (74, 75) and chronic kidney disease (48, 76), both comorbidities that are commonly seen in HFpEF patients, however SkM fibrosis in HFpEF has not been described. In our study, HFpEF mice demonstrated increased SkM fibrosis, accompanied by an increase in *Col1a1* and *Ctgf* gene expression. Although, no difference in these genes were seen in the human HFpEF SkM biopsy samples, FSP-1 protein expression, an indicator of the myofibroblast population, was increased in the SkM of both HFpEF mice and HFpEF patients. While the origin and role of myofibroblasts in SkM is unknown, studies show that myofibroblasts differentiate from fibro/adipogenic progenitors (47, 48). Additionally, type-1 pericytes also express FSP-1 and are activated in injured, diseased, and aged SkM (77), suggesting that all these cells could be activated in SkM in HFpEF. This is an important area of future investigation. As such, future studies should consider examining the involvement of these cell types in SkM fibrosis.

Finally, it has also been suggested that central mechanisms may play a pathophysiological role in SkM dysfunction in HF. For example, HF-related myopathy has been associated with increased activity of the ergoreflex which contributes to increased dyspnea with exertion, tachycardia, and the sensation of breathlessness via autonomic pathway (78, 79). Interestingly others have shown that in older patients with HFpEF, the autonomic reflexes controlling exercise heart rate are not compromised and as such do not contribute to the observed chronotropic incompetence (80).

### Limitations

In our study, we investigated the oxidative-predominant SkM and thus *soleus* muscle was studied in HFpEF mice as its oxidative nature is comparable to the oxidative-predominant human *vastus lateralis* muscle (16). Additional studies are warranted to compare other muscle types, such as glycolytic-predominant, in pre-clinical HFpEF models and HFpEF patients. In the present study, decreased capillary density and increased gene expression of collagen isoforms and *Ctgf* were seen in the *soleus* muscle of HFpEF mice, however, we did not observe changes in capillary density (16) nor changes in the collagen gene expression in human HFpEF. It is possibly that differences in fibrosis observed between animal and human may be due to a patchy distribution of fibrosis, as only a small sample (∼10mg) was sampled from the SkM biopsy, whereas in contrast, the whole *soleus* muscle was studied. It is also possible that this difference may also be reflective of the disease stage. The human HFpEF patients in our study were a stable, ambulatory cohort where > 80% of patients were NYHA Class II (16). Our cohort is similar to a cohort studied by Haykowsky et al., where adipose distribution and exercise intolerance was studied in obese HFpEF (57). In contrast, the *SAUNA* mice in this study represent more advanced HF disease as they demonstrate lung congestion, decrease exercise capacity, and elevated circulating natriuretic peptides (37, 38, 41–44), amongst other signs, all comparable with acutely decompensated HF patients. Circulating VEGF-α levels were measured and compared to a small cohort of control subjects and may require additional confirmation, although elevated VEGF-α levels was seen in the large PROMIS-HFpEF human study (31). Finally, the control subjects for the muscle biopsy experiments were not matched to the HFpEF patients (16) with regard to age, sex, race and BMI. The challenges remain recruiting older, healthy controls without cardiovascular disease for an invasive procedure. Additional studies are warranted to recapitulate the present findings.

**FIGURE 5:**
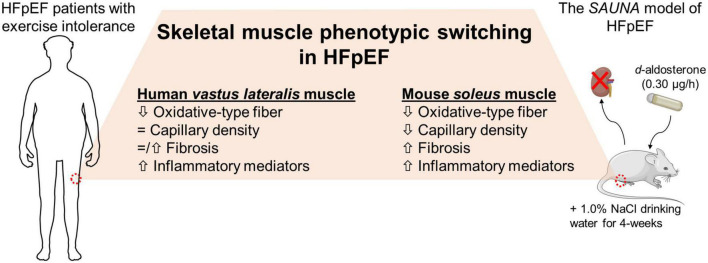
The HFpEF mouse model recapitulates the switch in SkM phenotype seen in patients with HFpEF. SkM phenotypic changes are evident in the HFpEF *SAUNA* mice. Comparison of molecular alterations between the HFpEF *SAUNA* mice and SkM biopsies from patients with HFpEF show that this SkM phenotypic switching is relevant and comparable to human HFpEF.

In conclusion, SkM phenotypic switching is seen in mice with HFpEF and is comparable to the SkM phenotype seen in patients with HFpEF (Figure 5). Our findings indicate that this pre-clinical model is a valuable tool to understand the role of SkM in HFpEF and facilitate the development of SkM-targeted therapy for HFpEF.

## Data availability statement

The original contributions presented in this study are included in the article/Supplementary material, further inquiries can be directed to the corresponding author.

## Ethics statement

The studies involving human participants were reviewed and approved by the University of Pennsylvania and Boston University Medical Campus Institutional Review Boards approved the human studies. The patients/participants provided their written informed consent to participate in this study. The animal study was reviewed and approved by the Institutional Animal Care and Use Committee at Boston University Medical Campus.

## Author contributions

ES, MV-M, and FS conceptualized the study, interpreted the data, wrote the first draft of the manuscript. PZ and JC provided human *vastus lateralis* muscle biopsy samples, and relevant clinical data. FS provided blood samples and relevant clinical data on HFpEF patients. MV-M and ES performed the surgeries. LW performed the physiological measurement. ES and LW performed the molecular analysis. All authors contributed to manuscript revision, read, and approved the submitted version.
