# Supplementary material for: Skeletal muscle phenotypic switching in heart failure with preserved ejection fraction
Source: Front Cardiovasc Med. 2022 Dec 1;9:1016452. doi: 10.3389/fcvm.2022.1016452 (PMC9753550; doi:10.3389/fcvm.2022.1016452)
Supplement: Supplementary file 1 [file Data_Sheet_1.pdf]

Supplementary Material

1 Supplementary Materials and Methods

UPenn and BMC HFpEF patients' inclusion and exclusion criteria:

Inclusion criteria for HFpEF patients: ***(1)*** Symptomatic HF (NYHA Class II/III) with a preserved EF (≥50%); ***(2)*** At least one month of stable medical management; ***(3)*** Elevated filing pressures which included at least one of the following: *(a)* history of elevated invasively determined filling pressure (i.e., left ventricular end diastolic pressure >16 mmHg, or, pulmonary capillary wedge pressure >15 mmHg); *(b)* use of intravenous diuretics in prior admission for HF; *(c)* mitral E/septal e' ratio >15; *(d)* mitral E/e' ratio >8 in addition to at least one of the following *(a)* Elevated NT-pro-BNP, *(b)* or chronic use of loop diuretics, *(c)* or left atrial volume index >34 mL/m^2. Control subjects were individuals who did not have a history of HF or hypertension. While other cardiovascular conditions were exclusionary, treated hypercholesterolemia was allowed in the control group to allow representation of elderly subjects.

Exclusion criteria: ***(1)*** Current atrial fibrillation; ***(2)*** Inability to exercise; ***(3)*** Cardiac diseases such as known hypertrophic, inflammatory or infiltrative cardiomyopathy, pericardial disease, current angina due to clinically-significant obstructive epicardial coronary disease and acute coronary syndrome within the past two months; ***(4)*** Moderate or greater aortic or mitral valve disease; ***(5)*** ischemia on stress-testing without subsequent revascularization or demonstration of non-obstructive epicardial coronary disease on coronary angiography; ***(6)*** Primary pulmonary arterial hypertension and uncontrolled hypertension (>180/110 mmHg at baseline); ***(7)*** Liver disease impacting synthetic function or volume control; ***(8)*** eGFR<30 $mL/min/m^2$ or Creatinine>2.5 mg/dL; ***(9)*** significant lung disease; ***(10)*** Hemoglobin < 10 g/dL; ***(11)*** Alcohol dependence or chronic narcotic use.

Human *vastus lateralis* muscle biopsy (UPenn Cohort):

Prior to the SkM biopsy procedure, subjects were stable for the past 30 days and were asked to avoid strenuous activity for 48 hours. Subjects arrived at the research unit in fasted state. The lateral aspect of the thigh, approximately half the distance between the iliac crest and the patella, was sterilized and anesthetized using lidocaine with sodium bicarbonate. A 5 mm skeletal muscle biopsy needle (Millennium Surgical) was introduced into the *vastus lateralis*, and biopsies were taken following the application of suction (1).

Human serum samples for VEGF-α analysis (BMC Cohort):

Blood samples were obtained from patients with chronically stable HFpEF in the ambulatory HF Clinic at Boston Medical Center. Samples were centrifuge at 2,000g for 15 min within one hour from collection, aliquoted and store at -80 °C.

Immunofluorescent analysis:

Mice *soleus* muscle was embedded in Tissue-Plus™ O.C.T. compound (Fisher Scientific, 4585), frozen in liquid nitrogen-cooled isopentane (Fisher Scientific, O3551-4) and stored at -80°C. The muscles were cut into seven micrometer thick sections with a cryostat (Leica, CM3050S) maintained at -20°C.

There are 4 major myosin heavy chain (MHC) isoforms in mouse SkM: ***(1)*** MHCI in type-1 slow twitch oxidative fiber, ***(2)*** MHCIIa in type-2A fast twitch oxidative fiber, ***(3)*** MHCIIx in type-2X fast twitch glycolytic fiber, ***(4)*** MHCIIb in type-2B fast twitch glycolytic fiber (2). To examine the fiber composition, mice muscle sections were first pre-treated with a cocktail containing Fab fragments anti-mouse IgG (Jackson ImmunoResearch, 115-007-003; 1:10) and anti-mouse IgM (Jackson ImmunoResearch, 115-007-020; 1:10) for two hours to block endogenous immunoglobulins and to reduce background staining. Then, muscle sections were incubated with a cocktail containing primary antibodies against MHCI (Development Studies Hybridoma Bank (DSHB), BA-D5; 1:100), MHCIIa (DSHB, SC-71; 1:100), MHCIIx (DSHB, 6H1; 1:50) and dystrophin (Invitrogen, PA5-323888; 1:100; SkM-specific marker) sequentially. Secondary antibodies were anti-rabbit IgG conjugated with Alexa Fluor™ 488 (Invitrogen, A11008; 1:200), anti-mouse IgG1 conjugated with Alexa Fluor™ 568 (Invitrogen, A21124; 1:200), anti-mouse IgG2b conjugated with Alexa Fluor™ 647 (Invitrogen, A21242) and anti-mouse IgM conjugated with DyLight™ 405 (Jackson ImmunoResearch,115-475-075; 1:100). Unstained fiber was identified as type-2B. DAPI (Santa Cruz, SC3598, 1:1,000) was used nuclear staining. The muscle sections were mounted with Fluoromount-G medium (Invitrogen, 00-4958-02). The specificity of primary antibodies against MHCI, MHCIIa and MHCIIx were individually tested on mouse gastrocnemius muscle, heart, and kidney (**Supplementary Figure S2**).

To examine the density of capillaries, sections were probed with Isolectin GS-IB$_4$-AlexFluor™ 647 conjugate (Invitrogen, I32450; 1:200) and anti-dystrophin antibody (Invitrogen, PA5-323888; 1:100). Anti-rabbit IgG secondary antibody conjugated with Alexa Fluor™ 488 (Invitrogen, A11008; 1:200) was used.

All images were captured at 10X using Leica SP5 confocal microscope. Image J software (National Institutes of Health) was used for image analysis.

Picrosirius red analysis:

To examine the levels of fibrosis, mice muscle sections were stained with 0.04% Fast Green (Sigma-Aldrich, F7258) in 1.3% saturated picric acid (Sigma-Aldrich, 197378) for 30 mins and followed by 0.1% Fast Green /0.04% Sirius red (Sigma-Aldrich, 365548) in 1.3% saturated picric acid for 30 mins(3). Then, the muscle sections were dehydrated in 50% ethanol, 70% ethanol, 90% ethanol, 100% ethanol and xylene sequentially for three minutes. The muscle sections were mounted with VectaMount® permanent mounting medium (Vector laboratories, H-

5000). The images were captured at 20X using a BZ-9000 BioRevo microscope (Keyence). Adobe Photoshop software (Adobe Inc.) was used for image analysis. The fibrotic area was normalized to total tissue area and expressed as fold change relative to the Sham mice.

Gene expression analysis:

Mice tissues: Total RNA was extracted from *soleus* muscle using RNeasy® Plus Universal Mini kit (Qiagen, 73404). DNase treatment (Invitrogen, AM2222) was performed on 1.5 µg of total RNAs for 40 mins at 37 °C. Then, 0.8 µg of DNase-treated RNA was reverse transcribed using High-Capacity cDNA Reverse Transcription kit (Applied Biosystems, 4368814). The reaction mixture was incubated at 25 °C for 10 mins, 37 °C for 120 mins and finally at 85 °C for 5 mins. Quantitative polymerase chain reaction (qPCR) was performed with PerfeCta SYBR® Green FastMix (Quanta Biosciences, 95074-012) using ViiA7 PCR system (Life Technologies). The reaction mixture was incubated at 95 °C for 10 mins for initial denaturation, followed by 40 cycles of denaturation at 95 °C for 15 seconds and primer annealing at 60 °C for 60 seconds. After amplification, PCR amplicons were subjected to the gradual increment of temperature for melt curve analysis.

Human tissues: Total RNA was extracted from *vastus lateralis* muscle using RNeasy® Plus Universal Mini kit (Qiagen, 73404). 150 ng of total RNA was treated with DNase (Invitrogen, AM2222) and followed by reverse transcription using High-Capacity cDNA Reverse Transcription kit (Applied Biosystems, 4368814). Then, cDNA was pre-amplified using TaqMan® PreAmp Master Mix (Applied Biosystems, 4391128) and a pooled TaqMan® probes (**Supplementary Table 2**). The reaction mixture was incubated at 95 °C for 10 mins for enzyme activation, followed by 14 cycles of denaturation at 95 °C for 15 seconds and primer annealing at 60 °C for 4 mins, and enzyme inactivation at 99 °C for 10 mins. Quantitative polymerase chain reaction (qPCR) was performed with TaqMan® Fast Advanced Master Mix (Applied Biosystems, 4444963) using ViiA7 PCR system (Life Technologies). The reaction mixture was incubated at 50 °C for 2 mins for UNG incubation, 95 °C for 2 seconds for enzyme activation, followed by 40 cycles of denaturation at 95 °C for 1 second and primer annealing at 60 °C for 20 seconds. For the measurement of *IFNG* gene expression, only four control subjects and seven HFpEF patients' samples showed positive amplification while the expression of *IFNG* was undetermined in the rest of the samples.

Both mouse-specific (Sigma-Aldrich) and human-specific primers (Applied Biosystems) were pre-designed and -validated. The details of primers are provided in **Supplementary Table 1 and 2**. For gene expression analysis of mouse tissue, the expression of gene of interest was normalized to the expression of hypoxanthine phosphoribosyltransferase *(Hprt)*. For gene expression analysis of human tissue, the expression of gene of interest was normalized to the expression of *18S*. All data was analyzed using ΔΔ Ct method and expressed as fold change relative to the respective control group.

Western blot analysis:

Mice *soleus* muscle and human *vastus lateralis* muscle were homogenized in ice-cold RIPA buffer (150 mM NaCl, 0.1% sodium dodecyl sulfate solution, 1% Triton X-100, 1% sodium deoxycholate in 50 mM Tris-HCl, pH 8.0) followed by centrifugation to obtain clear protein lysates. The lysates were resolved by sodium dodecyl sulfate (SDS)-polyacrylamide gel electrophoresis (PAGE) and transferred onto polyvinylidene fluoride (PVDF) membrane (Merck Millipore, IPVH00010). Membranes were probed with primary antibodies overnight (**Supplementary Table 3**). After overnight incubation, the membranes were probed with respective horseradish peroxidase (HRP)-conjugated secondary antibodies (**Supplementary Table 3**) for one hour at room temperature. Membranes were incubated with ECL substrate reagent (BioRad, 1705060) and the chemiluminescence signal was captured by ImageQuant™ LAS imaging system. Band density was analyzed using Image J software (National Institutes of Health). Band intensity of protein of interest was normalized to the band intensity of α-/β-TUBULIN and expressed as fold change relative to the control group.

Power Calculation:

To determine the sample size for mice experiments, the power analysis used $\beta=0.2$ (80% power) and $\alpha=0.05$ for the detection of a 25% expression change in the genes and proteins of interest. These calculations, as well as our observations based on the mortality rate of HFpEF mice in the preliminary experiments, indicated that 5-10 mice per group was needed to detect significant changes. Similarly, based on our preliminary analyses on human muscle samples (UPenn cohort), power analysis showed that 9-10 samples per group was needed to detect 25% expression change of gene and protein of interest using $\beta=0.2$ (80% power) and $\alpha=0.05$. Additionally, the power analysis showed that at least 3 controls and 15 HFpEF samples were needed to detect 15% changes in circulating VEGF- α level in the human serum cohort (BMC cohort) using $\beta = 0.2$ (80% power), $\alpha = 0.05$ at an enrollment ratio of 1:5 (control:HFpEF).

2 Supplementary Tables

Supplementary Table 1: Mouse-specific primers for gene expression analysis

Gene	Forward primer	Reverse primer	Amplicon size (bp)
Ndufa4	TGTTTAATCCAGATGTCAGC	TGTAGTCCACATTCACAGAG	105
Ndufb7	ACTAAAGAAGGGTAAGGCAG	CATCATCTCTTGTTGTGTGG	168
Ndufb8	CATGTGTAAACATCTCTTCGG	TCCTCAGATATCATAGTGAACC	181
Cox7c	GTAGAAAGGGGAGTTAGGTG	GAAAATGGCAAATTCTTCCC	168
Slc25a4	ATCATCATCTACAGAGCTGC	ATAATATCAGCCCCTTTCCG	200
Col1a1	CGTATCACCAAACTCAGAAG	GAAGCAAAGTTTCCTCCAAG	183
Col3a1	ACTCAAGAGTGGAGAATACTG	AACATGTTTCTTCTCTGCAC	166
Ctgf	GAGGAAAACATTAAGAAGGGC	AGAAAGCTCAAACTTGACAG	75
Il1b	GGATGATGATGATAACCTGC	CATGGAGAATATCACTTGTTGG	163
Il6	CCAGAGATACAAAGAAATGATGG	ACTCCAGAAGACCAGAGGAAAT	88
Ifng	TGAGTATTGCCAAGTTTGAG	CTTATTGGGACAATCTCTTCC	159
Tnf	CCCTCACACTCAGATCATCTTCT	GCTACGACGTGGGCTACAG	61
Ccl2	CAAGATGATCCCAATGAGTAG	TTGGTGACAAAAACTACAGC	87
Hprt	AGGGATTTGAATCACGTTTG	TTTACTGGCAACATCAACAG	116

Supplementary Table 2: Human-specific primers for gene expression analysis

Gene	TaqMan™ Assay ID	Amplicon size (bp)
NDUFA4	Hs00800172_s1	80
NDUFB7	Hs00958815_g1	58
NDUFB8	Hs00428204_m1	85
COX7C	Hs01595220_g1	123
SLC25A4	Hs00154037_m1	85
COL1A1	Hs00164004_m1	66
COL3A1	Hs00943809_m1	65
CTGF	Hs00170014_m1	60
IL1B	Hs01555410_m1	91
IL6	Hs00174131_m1	95
IFNG	Hs00989291_m1	73
TNF	Hs00174128_m1	80
CCL2	Hs00234140_m1	101
18S	Hs03003631_g1	69

Supplemental Table 3: Primary and secondary antibodies for western blot analysis

Antibody	Host	Dilution	Molecular weight (kDa)	Manufacturer / Catalogue number
Anti-VEGF-α	Goat	1:1,000	24	Santa Cruz / SC-1836
Anti-KDR	Rabbit	1:1,000	150	Invitrogen / PA5-16487
Anti-FSP-1	Rabbit	1:1,000	12	Cell signaling technology / 13018
Anti-α-/β-TUBULIN	Rabbit	1:5,000	50,52	Cell signaling technology / 2148
Anti-goat HRP conjugated	Rabbit	1:3,000	-	R&D systems / HAF017
Anti-rabbit HRP conjugated	Goat	1:3,000	-	R&D system / HAF008
Anti-mouse HRP conjugated	Goat	1:3,000	-	Invitrogen / J1718

3 Supplementary Figures

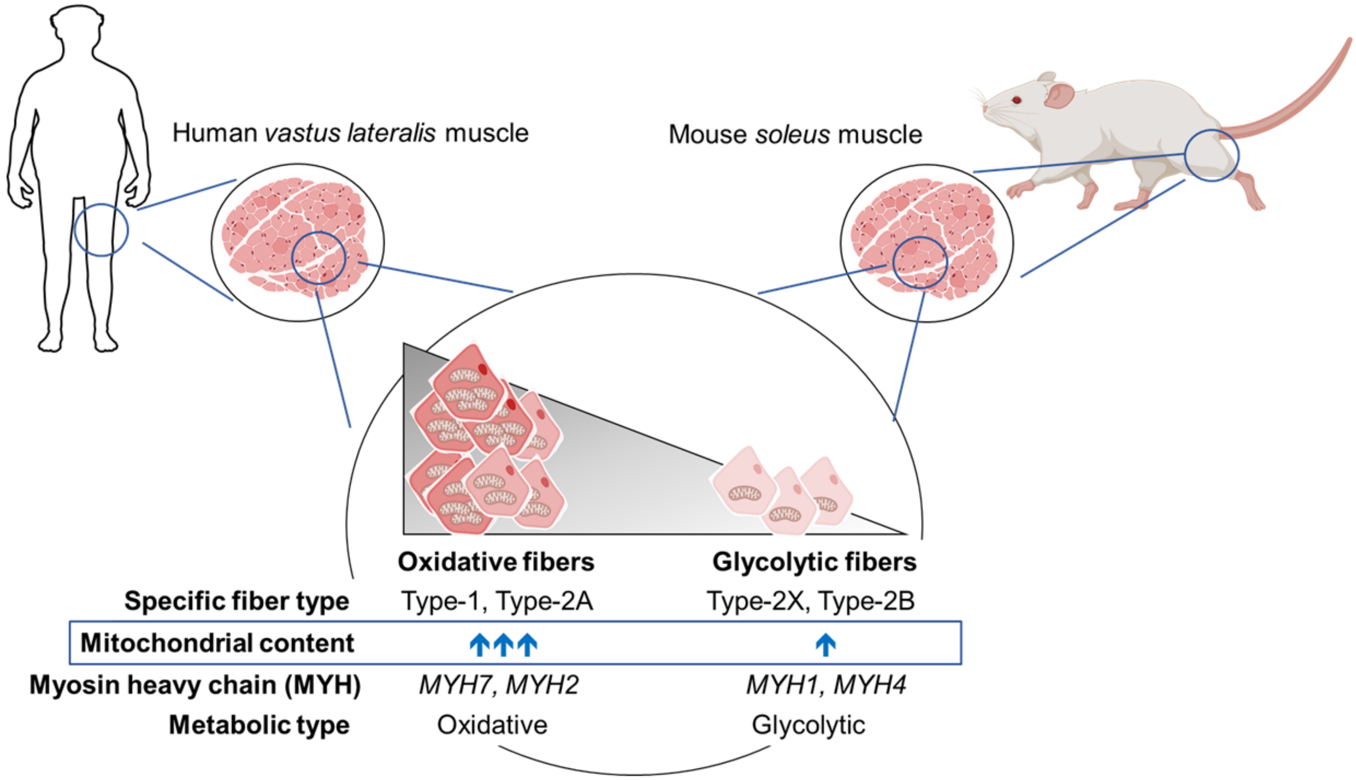

Supplemental Figure S1. Muscle fibers in human vastus lateralis and mouse soleus muscle. The human vastus lateralis and mouse soleus muscles are rich in type-1 and type-2A oxidative fibers. The type-1 and type-2A oxidative fiber are characterized by abundant mitochondria and the expression of myosin heavy chain (*MYH*) *7* and *MYH2*, respectively. Furthermore, the type-2X and type-2B glycolytic fibers are sparse in mitochondria and are identified by *MYH1* and *MYH4* expression, respectively.

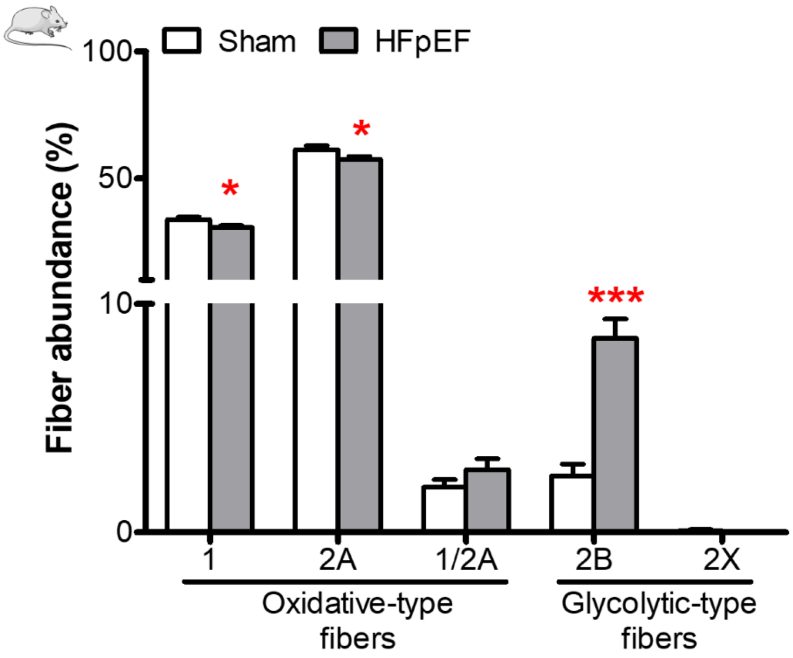

Supplementary Figure S2. Fiber composition in soleus muscle in Sham and HFpEF mice. Quantitative analysis shows a significant decrease in the fiber abundance of type-1 (30.7 ± 0.8 *vs.* 33.8 ± 1.0 % in Sham; P<0.05), type-2A (57.7 ± 1.0 *vs.* 61.4 ± 1.5 % in Sham; P<0.05) and a significant increase in the fiber abundance of type-2B (8.4 ± 0.8 *vs.* 2.5 ± 0.5 % in Sham; P<0.0001) in *soleus* muscle of HFpEF mice (n=11) *vs.* Sham mice (n=7). There were no significant changes in the fiber abundance of type-2X (0.04 ± 0.01 *vs.* 0.07 ± 0.06 % in Sham) and type-1/2A hybrid (2.7 ± 0.5 *vs.* 2.0 ± 0.3 % in Sham) in *soleus* muscle of HFpEF and Sham mice. All data are presented as mean ± SEM. Statistical analysis by Student *t* test for normally distributed data or Mann-Whitney *U* test for non-distributed data. *P<0.05; ***P<0.001 *vs.* Sham mice.

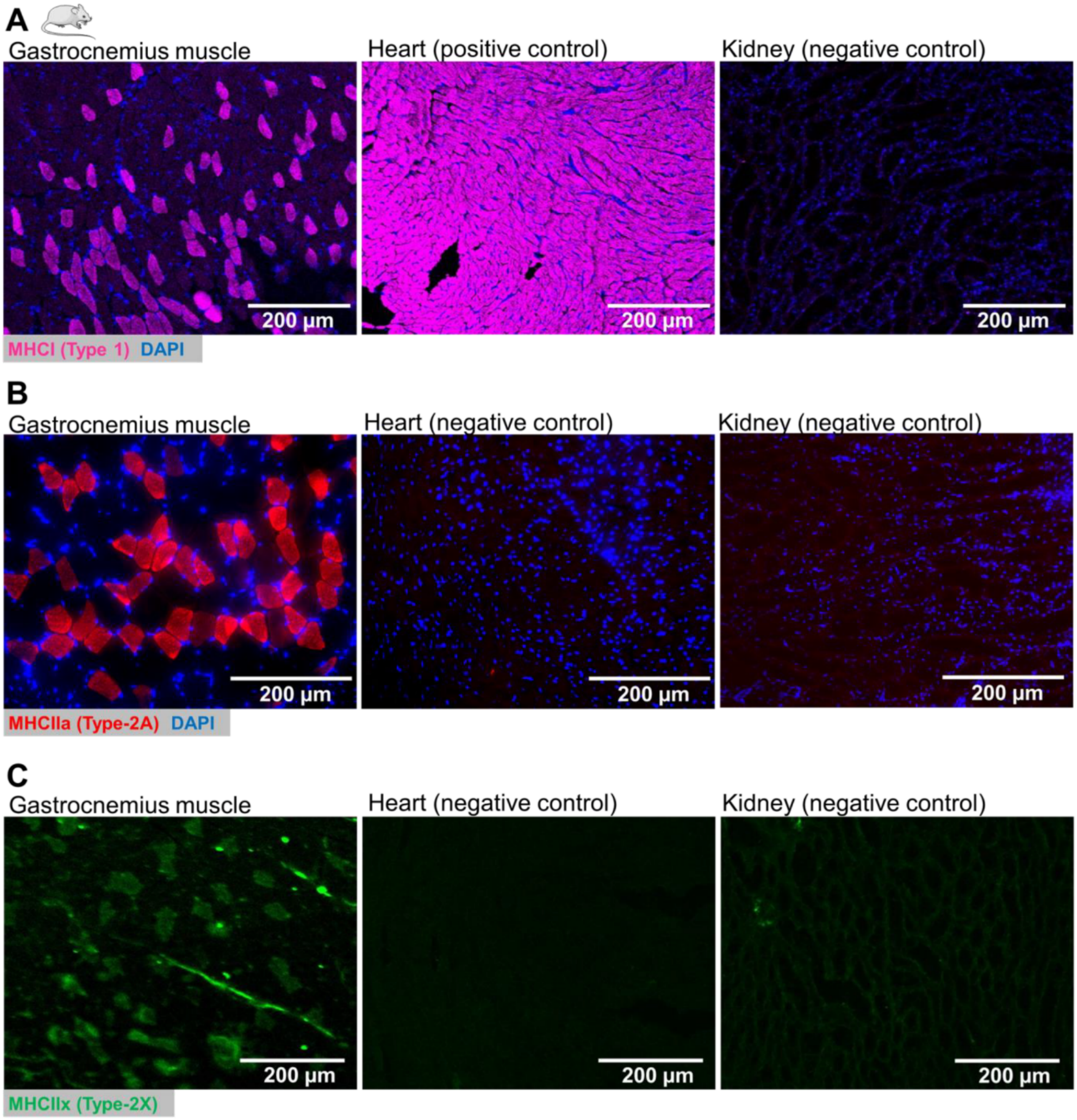

Supplementary Figure S3. Examination of the specificity of primary antibodies against MHCI, MHCIIa and MHCIIx in Mice. Representative fluorescent images showing **(A)** positive MHCI staining in gastrocnemius muscle, heart (positive control) and negative staining in kidney (negative control) of C57BL/6J mouse. **(B)** Positive MHCIIa and (**C**) MHCIIx staining in gastrocnemius muscle and negative staining in both heart and kidney (negative control) of C57BL/6J mouse.

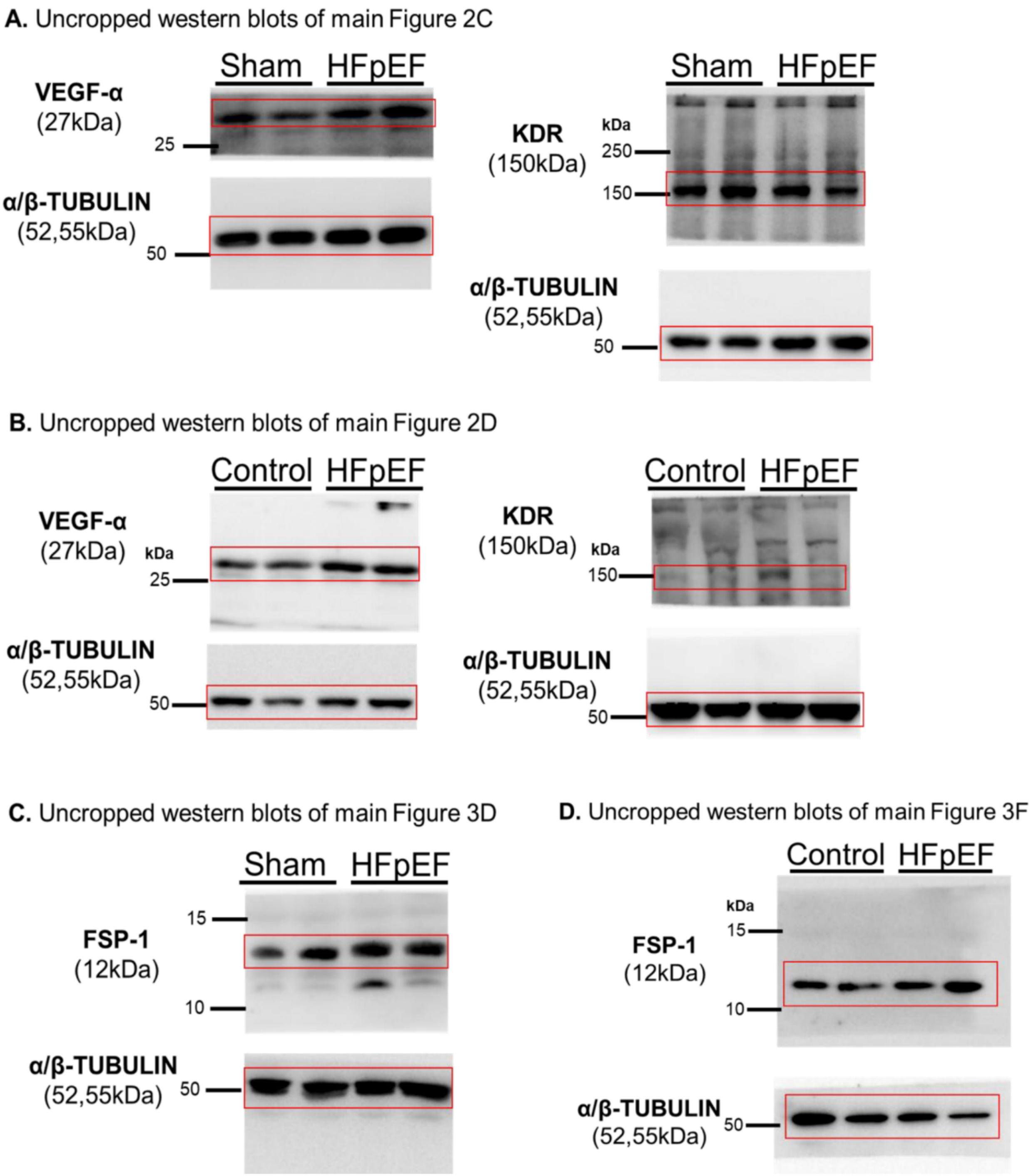

Supplementary Figure S4. **Uncropped blots (A)** Uncropped blots of VEGF-α and KDR of Sham and HFpEF mice shown in Figure 2C. **(B)** Uncropped blots of VEGF-α and KDR of control subjects and HFpEF patients shown in Figure 2D. **(C)** Uncropped blots of FSP-1 of Sham and HFpEF mice shown in Figure 3D. **(D)** Uncropped blots of FSP-1 of control subjects and HFpEF patients shown in Figure 3F.

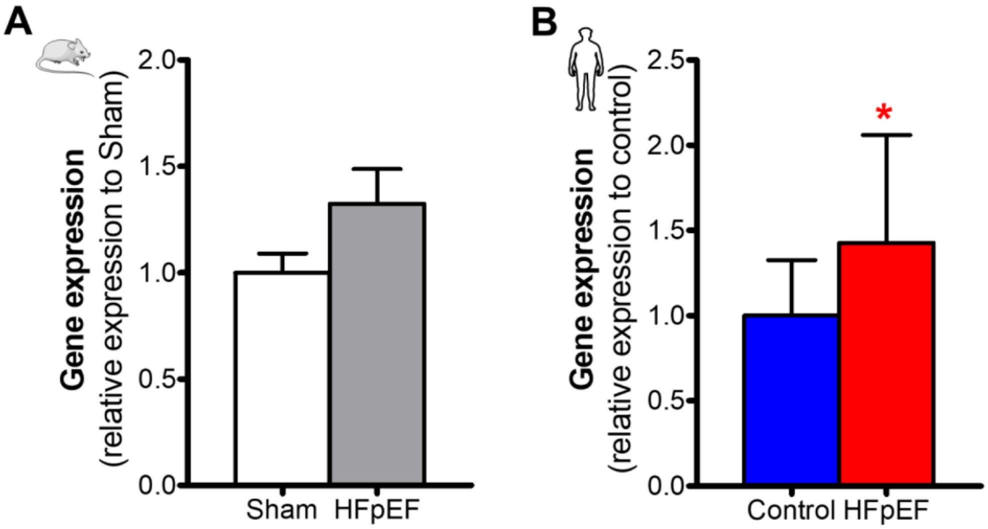

Supplementary Figure S5. Gene expression of hypoxia inducible factor 1 alpha subunit (HIF1A) in SkM of HFpEF mice and patients with HFpEF. (A) In mice, quantitative analysis shows that *Hif1a* gene expression in *soleus* muscle was not significantly different between HFpEF mice and Sham (1.3 ± 0.2 in HFpEF [n=15] *vs.* 1.0 ± 0.1 in Sham [n=7]). **(B)** In human, quantitative analysis shows that *HIF1A* gene expression was significantly increased in *vastus lateralis* muscle of HFpEF patients (1.4 ± 0.6; P<0.05; n=11) *vs.* control (1.0 ± 0.3; n=13). Statistical analysis by Student *t* test for normally distributed data or Mann-Whitney *U* test for non-distributed data Mouse and human data are presented as mean ± SEM and mean ± SD, respectively. *P<0.05 *vs.* control subjects.

4 Supplementary References

1. Tarnopolsky MA, Pearce E, Smith K, Lach B. Suction-Modified Bergström Muscle Biopsy Technique: Experience with 13,500 Procedures. *Muscle Nerve* (2011) 43(5):717-25. Epub 2011/04/05. doi: 10.1002/mus.21945.

2. Talbot J, Maves L. Skeletal Muscle Fiber Type: Using Insights from Muscle Developmental Biology to Dissect Targets for Susceptibility and Resistance to Muscle Disease. *Wiley Interdiscip Rev Dev Biol* (2016) 5(4):518-34. Epub 2016/05/21. doi: 10.1002/wdev.230.

3. Segnani C, Ippolito C, Antonioli L, Pellegrini C, Blandizzi C, Dolfi A, et al. Histochemical Detection of Collagen Fibers by Sirius Red/Fast Green Is More Sensitive Than Van Gieson or Sirius Red Alone in Normal and Inflamed Rat Colon. *PLoS One* (2015) 10(12):e0144630. Epub 2015/12/18. doi: 10.1371/journal.pone.0144630.
